# Effect of Nicotine on Immune System Function

**DOI:** 10.34172/apb.2023.008

**Published:** 2022-01-04

**Authors:** leila Mahmoudzadeh, Seyyed Meysam Abtahi Froushani, Marjan Ajami, Maryam Mahmoudzadeh

**Affiliations:** ^1^Division of Immunology, Department of Microbiology, Faculty of Veterinary Medicine, Urmia University, Urmia, Iran.; ^2^Department of Food and Nutrition Policy and Planning Research, Faculty of Nutrition Sciences and Food Technology, National Nutrition and Food Technology Research Institute, Shahid Beheshti University of Medical Sciences, Tehran, Iran.; ^3^Nutrition Research Center and Department of Food Science and Technology, Faculty of Nutrition and Food Science, Tabriz University of Medical Sciences, Tabriz, Iran.; ^4^Drug Applied Research Center, Tabriz University of Medical Sciences, Tabriz, Iran.

**Keywords:** Nicotine, Immunomodulation, Autoimmune disease, Cancer

## Abstract

As a parasympathetic alkaloid and the main substance in cigarette smoke, nicotine modulates the immune system, inhibits innate and acquired immunity and is used in treating many autoimmune diseases. It often stimulates the α7 receptor and causes an anti-inflammatory state in the body. This study is designed to evaluate the role of nicotine treatment on immune system. The results showed that nicotine affects many cells in immune system, alters the downstream intracellular mechanisms and changes lymphocytes polarization. This substance alters TLRs and STATs gene expression and thus changes in the innate immune system. All these events inhibit the secretion of pro-inflammatory cytokines and chemokines which increase angiogenesis and metastasis and exacerbates tumors due to increasing survival and cell growth. Nicotine can aggravate tumors in cancer patients, with many positive effects observed in the treating autoimmune disease, Nicotine treatment function in different conditions depends on factors such as concentration, how it is employed, treatment duration and other conditions such as body conditions affecting the immune system, hence, further studies and review of all conditions are required.

## Introduction

 Smoking is amongst unhealthy behaviors that endangers public health and can impose significant costs on the society.^1^ Smoking is the most important cause of death in 35-69 year-old individuals in developing countries.^2^ In Iran, the majority of smokers (66.7%) had their first cigarette at age the 14. According to national reports, the prevalence of smoking ranged between 3.8% and 30.1% in different cities and had a total prevalence of 14.3% in Iran, which was significantly higher in males than females.^3^ Smoking is a major cause of cancer, cardiovascular disease, lung disease and death.^4^ It also delays wound, fracture, and bone healing.^5^ The chemicals in cigarette smoke are released in two phases: particles and steam. Interestingly, secondhand smokers’ chronical exposure to smoke and cigarette particles suppresses the function of the immune system, while their chronic exposure to the vapor particles phase does not suppress immunity.^6^ Smoking also increases the risk of tuberculosis as well as the severity of the other infectious disease.^7^ Among the 3500 different chemicals in cigarette smoke, nicotine is a major component. It is a parasympathetic alkaloid found in high doses in tobacco plant leaves and in small amounts in other plants such as the *Solanaceae* family. This compound is also present in the formula of many pesticides. In addition, nicotine is the main ingredient of cigarettes and is a major cause of dependence on cigarettes.^8^ On average, each cigarette contains 14-10 mg of nicotine.^9^ Nicotine acts as a cholinergic substance. It stimulates the release of acetylcholine either directly by stimulating nicotinic or muscarinic receptors or indirectly by inhibiting cholinesterase or other mechanisms.^10^ Nicotine appears to play the role as an inhibitor of immune function and inhibits the secretion of many cytokines.^11^

 It seems that despite the completely destructive and harmful effects of smoking, nicotine, although one of the causes of cigarette smoke addiction, may also have beneficial effects such as immunomodulatory effects.^11^ Nicotine reduces T cell receptor (TCR) signaling and suppresses the production and secretion of antibodies.^12^ which explains the reduction in autoimmune diseases such as ulcerative colitis (UC) and sarcoidosis in smokers.^6^ All effects of nicotine seemingly depend on its concentration and how it is used.^11,13^ Nicotine appears to cause extensive changes in the immune function, increase in leukocyte count and TCD8^+^ accompanied by a sharp decrease in TCD4^+^ and NKs observed in smokers.^14^ In rats, subcutaneous or intra-cerebroventricular injection of nicotine reduces the secretion of T-dependent antibodies, the proliferation of T responses and inhibition of TCR pathway signaling.^15^

 Numerous studies are conducted on nicotine responses in cancer. Smokers with pancreatic and breast cancer have a worse prognosis compared to non-smokers.^16,17^ Furthermore, Nicotine alone does not appear to be carcinogenic; however, it induces cell proliferation and angiogenesis in various experimental models. Nicotine increases COX-2, prostacyclin, VEGFR-2, MMPs, uPA and e-NOS activity expression.^18,19^ Nicotine appears to have the ability to induce invasion, cell migration and tumor genesis in an a7-receptor and Src-dependent manner. Although there are many theories about the molecular mechanism of nicotine angiogenesis in tumors, the exact mechanism is still unclear.^18^

 Nicotine acetylcholine receptors (nAChRs) are expressed on the surface of different cells, including immune and nerve cells.^20^ Chronic nicotine exposure activates AChR. This receptor is an ion channel that is also activated by neuro-transmitters like acetylcholinein the body.^21^ nAChR is made of 17 subunits in mammals including (α1-α7, α9, α10, β1-β4, γ, δ and μ) that are encoded by 17 different genes.^22,23^ A subunit of nAChRs types is discovered in immune cells. Other cells also have different combinations of nicotine receptor subunits, so each cell’s response to nicotine is different.^21,24^

 Generally, this study aimed to provide and summarize the available information on the immunomodulatory potential of nicotine and the need for research on specific agonists of α7nAChR.

## Cholinergic anti-inflammatory pathway

 The cholinergic anti-inflammatory pathway provides a deep connection between the nervous system and the immune system. The afferent vagus nerve fibers can detect injury, pathogens, and tissue ischemia and relay information to the motor centers of the vagus nerve.^25,26^ In the following, efferent vagus nerve fibers are activated and hamper cytokine production by macrophages and other innate cells through the release of acetylcholine. The vagus nerve terminals in the celiac ganglion connect to the splenic nerve, which uses norepinephrine (NE). NE binds to β2ARs on special CD4+ T cells, which in turn release acetylcholine to acetylcholine α7nAChR innate cells to promote an anti-inflammatory response.^25,27^

###  Effects of nicotine on innate immune system

 In a model of sepsis, nicotine inhibits inflammation induced by TLR4 stimulation and interferes with *α*7-nAChR receptors, increasing a person’s chances of survival.^28,29^ In the presence of LPS, Nicotine suppresses TLR4 expression in monocytes and tumor necrosis factor alpha (TNFα) production by peripheral blood mononuclear cells (PBMCs).^30^ All these activities can be inhibited by α7 antagonists such as mecamylamine and *α*-bungarotoxin. In the presence of LPS, all NF-κB and P38MAPK inhibitors can mimic nicotine function.^30^ The α7-nAChR and nicotine agonists inhibit the secretion of inflammatory cytokines by PBMCs. This is done through inhibiting the JAK2-STAT3 pathway.^31^ Nicotine is able to inhibit IRF7 and IRAK4 and inhibit RIG1 and TLR3 and thus inhibit antiviral responses and the spread of virus in the body.^32^

###  Effects of nicotine on secretion of cytokines and chemokines

 Nicotine increases the secretion of inflammatory phase enzymes such as caspase-1 and cytokines such as IL-1β and IL-18.^33^ Nicotine also increases the expression of the NF-κB inflammatory gene and suppresses autophagy in macrophages. Twenty-four hours after nicotine injection, the expression level of TGF-β is greatly increased which, in turn, causes the cells to polarize towards Treg. High values also increase with increasing nicotine concentration from 0.1Mμ to 1Mμ.^34^ Nicotine plays the role of an inhibitor of immune function and inhibits the secretion of many cytokines. Innate immune system receptors such as TLR2 and TLR4 and NOD receptors are significantly reduced in smokers and the secretion of cytokines IL6, IL8, TNF-α, IL10 and the chemokines CCL-2, CCL-5, CXCL9 and CXCL10 is altered.^11^ Nicotine α7 receptor appears to be expressed on TCD4+ lymphocytes. Stimulation with nicotine in these receptors reduces T proliferation and activation of T lymphocytes. All of these events reduce the secretion of IL-17, IL-17F, IL-21, and IL-22 cytokines. Increased polarization toward Th2 also increases IL-4 production. Decreased T.bet expression and increased GATA-3 expression suppress Th1 and Th17 more. T lymphocytes with α7 - / - marker are not affected by nicotine. there is an assumption that repeated administration of nicotine may also cause a state of unresponsiveness and tolerance in the immune system.^12^

## Nicotine concentration effect on immune system and cells

 Nicotine exposure suppresses the immune system, makes anti-inflammatory effects, and reduces the secretion of inflammatory antibodies and cytokines, thereby reduces the activity of lymphocytes. However, exposure to high levels of nicotine (1μM and above), as seen in smokers, increases the risk of infectious and autoimmune diseases.^11^ Another interesting study conducted in 2013 examined mesenchymal stem cells (MSCs) and periodontal ligament stem cells (PDLSCs) responses at a concentration of 1μM of nicotine. The results showed that MSCs and PDLSCs treated with 1μM of nicotine had a significant ability to proliferate and survive compared to the control group, but it seemingly had lower migration ability, hence the ability to move, the speed of movement and response in these cells are inhibited. Nicotine, at a concentration of 1μM, reduces and blocks MSCs differentiation and significantly reduces the expression of PTK (involved in cell migration), RUNX2, ALPL and BGLAP (bone marrow differentiation) genes. Also, the expression of several miRNAs related to growth, migration, proliferation and anti-inflammatory agents at 0.5μM and 1μM doses of nicotine was investigated, and it seems that inhibition and suppression of immunity is sometimes ten times higher at concentrations of 1μM nicotine. At 0.5μM concentration, nicotine increases the secretion of anti-inflammatory cytokines and raises the differentiation of Th0 lymphocytes to Th2.^35^

 IL-4 and IL-10 cytokines are sharply increased in the mice treated with 1μM of nicotine, and IFN-γ cytokines are highly reduced. Severe polarization to Th2 in the mice treated with high doses of nicotine increases allergic and autoimmune diseases.^22^ Nicotine appears to have an independent effect on leukocyte activation in addition to the central effects on the pituitary-hypothalamic axis that release endocrine corticosteroids. Increasing nicotine concentration increases the expression of FASL genes and the expression of caspase-3 thus increases cell apoptosis, but these effects are not observed at concentrations below 100 μM. In fact, cell survival at concentrations below 100 μM remains untouched.^36^ On the other hand, higher doses of nicotine stimulate angiogenesis and tumorigenesis. Doses of nicotine in the range of M5-10 and M7-10 cause the expression of VCAM-1, ELAM-1, bFGF and MMP-2 genes, all of which are involved in tumor angiogenesis and angiogenesis.^37^ Cucina et al achieved similar results indicating that increasing nicotine concentration increased angiogenesis and tumor spread by increasing the expression of bFGF and TGF-β. The mitogenic effects of nicotine can be reversed by injecting anti-bFGF and Anti-TGF-β antibodies.^13^

**Figure 1:**
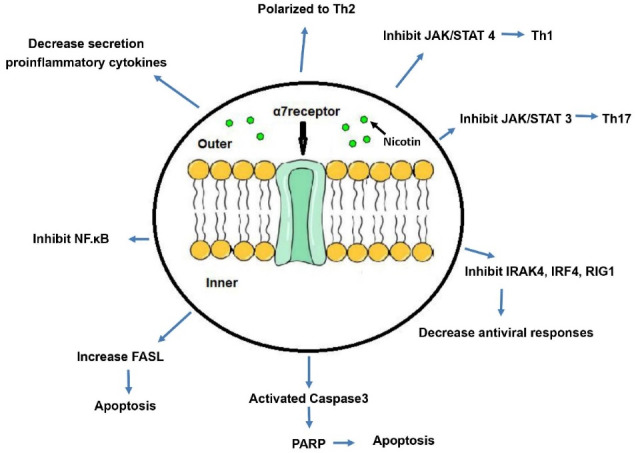
Effects of nicotine on various components of the immune system.Nicotine affects the alpha subunit of the nicotinic receptor on the phospholipid bilayer membrane and initiates intracellular cascading reactions. These reactions lead to major changes in the immune system. These cascading reactions inhibit inflammatory pathways by inhibiting NF-κB and inhibiting the secretion of inflammatory cytokines. On the other hand, they inhibit the polarization of Th0 lymphocytes to Th1 and Th17 and cause polarization to the non-inflammatory pathway of Th2. Inhibition of IRAK4, IRF4, and RIG1 expression also suppresses antiviral responses. Increased FASL receptor expression on the surface of immune cells as well as increased expression of caspase-3 leads to widespread apoptosis of immune cells. Abbreviations: JAK/STAT, Janus kinase/signal transducer and activator of transcription; Th, T helper; IRAK4, Interleukin-1 receptor associated kinase 4; IRF, Interferon regulatory factor 4; RIG1, retinoic-acid inducible gene I; PARP, poly-ADP ribose polymerase; FASL, Fas cell surface death receptor ligand; NF-κB, Nuclear factor kappa B.

 Chronic and acute stimulation of mice by nicotine suppresses the proliferative responses induced by Con.A. A single dose of 1mg/kg nicotine inhibits the proliferative responses of Con.A and PHA-induced T lymphocytes.^38,39^ Dose-dependent injection of nicotine increases circulating corticosterone levels and thus inhibits the immune responses.^39^ The effects of nicotine on various immune system components are shown in Figure 1.

 There are several nicotine receptors on human and rat CTL lymphocytes.^40^ Both TCD4^+^ and TCD8^+^ lymphocytes express anchor receptors on their surface. Stimulating CD3^+^ and CD28^+^ receptors alters the expression of these receptors on lymphocytes.^41^ Nicotine suppresses CTLs’ planning to become memory cells, but this is only seen in acute stimuli (for 3 days) and does not occur in chronic stimuli. Interestingly, the initial activation of TCD8^+^ lymphocytes remains intact, which is due to the lack of altered expression of the CD25, CD69, and CD44 genes three days after exposure to different concentrations of nicotine.^40^ This substance blocks the activation of endothelial cells and leukocyte implantation and suppresses the function of dendritic cells and polarization towards Th1.^42,43^

###  Effect of nicotine on autoimmune diseases and their treatment

 For several years, various studies have shown the link between smoking and autoimmune diseases.^44-46^ Cigarette smoke appears to produce free radicals, damage cell genome and trigger proteins to citrullination.^47^ Interestingly, the role of nicotine in autoimmune diseases, as a major component of smoking, has been debated. Whether nicotine has an inflammatory or anti-inflammatory effect on the body Is also debatable.^48^ The role of nicotine on different autoimmune diseases may be different.^46^

 Multiple sclerosis (MS) is a progressive disease that kills the immune system. Smoking is a risk factor for this disease and can exacerbate it.^49,50^ Subcutaneous administration of nicotine to EAE mice improved the disease and slowed down the demyelination process, while injecting cigarette reverses exacerbated disease. Nicotine suppresses microglial macrophages differentiation into M1 type and inhibits TNF-α secretion, while in the other group the components of cigarette smoke activated microglial macrophages and promoted EAE.^51^ Part of the anti-inflammatory effects of nicotine is due to the α7-nAChR subunit, while the other part is attributed to the α9 subunit. In EAE mice that α9 subunit is knocked out, there is a decrease in iNOS and IL-1β, which is independent of cholinergic stimulation.^46^

 Rheumatoid arthritis (RA) is an autoimmune disease with polarization disorder toward Th1 and Th2 that produces proinflammatory cytokines such as IL-1β, IL-6, IL-17, TNF-α, IFN-γ and IL-8 and, as a result, causes an inflammatory state in the body.^52-54^ Patients with RA have very low levels of cytokines secreted by Th2 lymphocytes.^52^ All of these events lead to involvement and destruction of the joints. Both environmental and genetic factors play a role in causing the disease,^55^ and smoking acts as an important environmental factor in causing this disease.^56^ Interestingly, nicotine, as a selective cholinergic agonist, promotes anti-inflammatory activity and disease modulation. Adding nicotine to the mineral water of experimental RA model mice four days before induction of the disease significantly reduces the amount of TNF-α and reduces the symptoms of the disease.^57^ In 2019, Golbahari and Abtahi Froushani showed that treatment with nicotine could reduce some of the hematological and biochemical parameters of rats with rheumatoid arthritis, like C-Reactive Protein, Rheumatoid factor, Myeloperoxidase, Nitric oxide, TNF-α, IFN-, γ IL-17, IL-6, and IL-1.^58^

 T1DM is an autoimmune disease defined by TCD4+ and TCD8+ lymphocytes invading Langerhans islet cells. This attack leads to the destruction of β cells and impaired insulin secretion in the body.^59^ During the disease, an increase in active inflammatory cytokines such as IL-12, IFN-γ, IL-1 and TNF-α is observed, while Th2-related cytokines such as Il-10 and IL-13 decrease. These events lead to an imbalance of Th1/Th2.^60^ Subcutaneous injection of nicotine at 0.4 mg/kg to experimental model mice with diabetes decreases the expression of Th1-related cytokines, Increases the expression of Th2-related cytokines in pancreatic islet cells. Also, at the end of week 25 after treatment, the disease worsened and the amount of insulin secretion increased.^61^

 Nicotine reduces the secretion of cytokines secreted by Th1 and Th17 such as IL-12p35, IL-6 and Il-23 and IL-1β and TNF-α in the experimental model of mice.^62^ Fibroblast-like cells in nicotine-treated RA patients are unable to secrete large amounts of TNFα, which appears to be cholinergic-dependent.^52^ However, Lindblad et al. showed almost contradictory results wherein spite of a decrease in the amount of IL-6 in the experimental model RA mice’ spleen, there was no visible decrease in the amount of TNF-α and, therefore, no symptoms of the disease.^62^

 The effects of nicotine on T1DM in diabetic retinopathy indicated increasing nicotine by 2.1 mg/kg per day for 9 weeks increased cataract scores, blood glucose, and inflammatory cytokines over time. This finding seems to contradict other nicotine-related studies.^63,64^

 Behçet’s disease is an inflammatory disease with systemic peripheral vascular inflammation, neutrophilic infiltration and inflammation of endothelial cells. The disease is associated with large and painful sores in the mouth and genitals, eye inflammation, skin lesions, joint pain and vasculitis With unknown pathogenesis.^65,66^ An increase in the number of Th1 cells and a disruption of the Th1/Th2 ratio is seen as the inflammatory cytokines of the Th1 class increase.^67^ In one study, the immunomodulatory effects of nicotine following keratinocyte cell treatment in patients with Behcet’s disease were demonstrated.^68^ There is also a report of successful treatment of a woman with oral ulcers caused by Behcet’s disease with nicotine treatment.^69^ Ciancio et al also reported that the skin wounds of 4 male patients with Behçet’s Disease were successfully treated with nicotine.^70^

 Sarcoidosis is an autoimmune disease that often affects the lungs with unknown cause which can also affect other parts of the body. In this disease, macrophages begin to secrete proinflammatory cytokines, leading to polarization toward Th1. This causes granulomatosis at the site of involvement.^71,72^ In 2013, nicotine was used to treat patients with acute sarcoidosis with pulmonary involvement. After 12 days of treatment, patients in stage 1 and 3 of lung disease did not show a clinical response to treatment, but an increase in T.reg levels and a decrease in TLR2 and TLR9 expression were observed. These receptors play a major role in the pathogenesis of granulomatous diseases such as sarcoidosis.^73^

 The group of inflammatory bowel diseases (IBDs) includes UC and Crohn’s disease which seem to depend on environmental and genetic factors. Impaired immune regulation in the gut leads to inflammatory cytokines and disease.^74,75^ Implantation and activation of intestinal monocytes and macrophages appear to play an essential role in the pathogenesis of the disease. Activation of these cells leads to the secretion of inflammatory cytokines such as (IL-1α, IL1β, IL-2, IL-6, IL-8, IL-12, IL-17, IL-23, TNF-α and IFNγ).^76^ In 1994, nicotine treatment was used for six weeks to treat UC which resulted in signs of complete recovery among 48.6% of patients, while 94.3% experienced side effects.^77^ Followed in 1997, and the results indicated that although nicotine reduces the symptoms of UC, it is not significantly different from standard immunosuppressive treatments for the disease,^78^ hence, the use of nicotine in the treatment of UC is doomed to failure.^79^ Studying patients with symptoms of UC and active intestinal autoimmune disease in 10 groups with nicotine treatment indicated side effects in 40% of the patients, and no improvement in histopathology, but 71% of patients showed relative improvement in clinical symptoms,^80^ which may be due to the binding of nicotine to the α7-nAChR receptor, the development of an anti-inflammatory state, and inhibition of inflammatory cytokines’ secretion in the intestinal wall.^81^

 Crohn’s disease is an inflammatory disease of the intestine with predominant gastrointestinal involvement, though it is sometimes associated with other autoimmune diseases and shows extra-intestinal symptoms.^82^ Findings on the effect of nicotine on these patients are contradictory.^83,84^ In a study on 10 patients with Crohn’s disease, nicotine treatment improved clinical symptoms and histopathology without side effects. However, the endoscopic symptoms of these patients were not evaluated while these symptoms must be evaluated, as well.^84^ Kikuchi et al suggested that chronic nicotine treatment exacerbates the disease by increasing Th1 levels. In mouse models of Crohn’s disease, nicotine treatment depends on the site of inflammation and the dose used on the colon and small intestine.^83^

**Figure 2:**
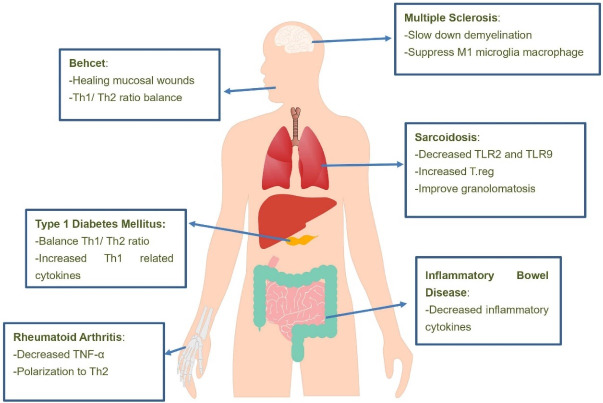
**
Mechanisms of nicotine on autoimmune diseases. ** It seems that treatment of patients with various autoimmune diseases with nicotine suppresses inflammatory pathways and ultimately improves and relieves the symptoms of these diseases. As shown in the figure, the effects of nicotine on patients with multiple sclerosis, sarcoidosis, inflammatory bowel disease, Behçet’s disease, type 1 diabetes mellitus and rheumatoid arthritis have been studied and various methods of reducing inflammation by nicotine in these patients have been shown. Abbreviations: TLR, Toll-like receptor; T.reg, T regulatory; Th, T helper; TNF-α, tumor necrosis factor alpha.

 Nicotine, as a major component of cigarette smoke and a cholinergic agonist, appears to inhibit inflammation and treat chronic IBDs.^45,53^ Smoking and cigarette smoke appear to predispose people to MS. However, nicotine alone is able to control the disease proven in the experimental animal model of EAE. Other compounds in cigarette smoke aggravate EAE/MS if nicotine is removed.^85^ Interestingly, brain microglia cells express many nicotine-related receptors and modulate nicotine immunity.^86^Figure 2 shows the mechanism of nicotine effects in various types of auto immune diseases.

###  Effects of nicotine on Cancers

 Given the toxicity of α7 antagonists and some conflicting studies in this field, it seems that targeting downstream effects of these receptors is a suitable alternative method, although more studies are required.^87^

 Nicotine plays a major role in regulating survival pathways and anti-apoptotic effects. Although cigarette smoke triggers tumor formation, it is possible that nicotine increases tumor survival by increasing cell survival and preventing cell apoptosis. Nicotine leads to resistance to treatment with chemotherapy drugsand radiation therapy for tumors.^88-90^ The phosphoinositide 3-kinase (PI3K)/AKT pathway is an important pathway activated through exposure to cigarette smoke and nicotine. The serine/threonine kinase AKT pathway is also a known pathway in cell cycle progression and cell survival. Nicotine appears to cause Akt path way phosphorylation.^91^ These effects are observed shortly after injection with different concentrations of nicotine (from low to high) and can be inhibited with PI3K inhibitor. Phosphorylated Akt is seen in lung tumors in smokers.^92^

 The effects of cell growth and survival in animal models are not inducible with mutant Akt, suggesting that increased nicotine-dependent cell growth is associated with Akt phosphorylation and stimulation.^91^ Survival induction by nicotine is mediated by the E2F1-dependent assay, suggesting that nicotine exposure can induce immunity to apoptosis through various mechanisms one of which is XIAP pathway.^93^ Nicotine also induces the MAPK pathway and thus NF-κB which is done by phosphorylation of ERK1/2 and degradation of NF-κB regulators called IκBα.^94^

 Stimulating α7 and α9 subunits of nicotine receptors exacerbates lung cancer, increases metastatic activity and cell division and increases tumor cell survival in pulmonary adenocarcinoma. These subunits play a major role in pulmonary adenocarcinoma.^95^ Interestingly, nicotine itself up-regulates the α7 nicotinic receptor on squamous cell lung cancer cells by activating the Sp1/GATA protein.^96^ In addition to affecting cell proliferation and survival pathways, nicotine is also involved in cell growth and metastasis pathways. The role of nAChRs in migration and metastasis in breast, pancreatic and lung cancers is proven.^97^ Nicotine is a potent inducer of epithelial-mesenchymal transition by inhibiting the expression of E-cadherin and β-catenin and can induce and facilitate transcription of mesenchymal genes such as fibronectin and vimentin in many cell lines.^87^ Nicotine causes metastasis and tumor spread by inducing the expression of the MMP15, MMP14 and MMP9 genes in an E2F1-dependent pathway.^98^ Nicotine treatment also increases the expression of CD44 and BMI1 proteins and exacerbates squamous cell carcinoma tumors.^99,100^ Nicotine plays a role in increasing the self-renewal capacity of stem cells and stem cell-like populations.

 Due to the many roles nAchRs play in increasing cell growth, metastasis and tumor growth, efforts were made to combat smoking-induced lung cancer,^101,102^ but the main concern about the use of nAchRs antagonists is their destructive effect on the neuromuscular system. In addition, many of the receptor antagonists are known as toxins, including α-cobratoxin and α-bungarotoxin in the venom. Interestingly, mouse models used these toxins in very small amounts to combat xenograft tumors, and no damage or functional reduction in neurological and brain activity was observed in these cases.^102^

###  Effects of nicotine on immunological tests

 Nicotine significantly reduced NO production and, in fact, reduced inflammation. The presence of a nicotine inhibitor such as Fenofibrate eliminates the effects of nicotine in inhibiting NO production. The same is true for NBT. The presence of nicotine in the vicinity of cells reduces the effects of phagocytosis and respiratory explosion and causes immunosuppressive and anti-inflammatory effects.^103^ In the case of neutral test (NR), nicotine is shown to be more effective than similar compounds and nicotine derivatives such as caffeine, niacinamide and nicotinic acid in most cell lines to reduce the rate of neutral cation depletion and cell inhibition.^104^

 In MTT test, no significant difference was observed between different groups treated with supernatant of MSCs with/without nicotine and between control groups because of nicotine concentration. As stated, the effects of nicotine proliferation and survival are not observed at concentrations below 100 μM. In fact, cell viability remains intact at concentrations below 100 μM nicotine.^36^ It suggested that higher concentrations of nicotine are required to induce apoptosis and cell death in different cell lines. Concentrations higher than 6 μM are required to observe the effect of nicotine on cell growth and proliferation and concentrations around 1 μM can also increase growth and proliferation.^105,106^

## Conclusion

 Despite the completely destructive and harmful effects of cigarette smoke, nicotine via stimulation of the α7 receptor can promote the anti-inflammatory benefits on the immune system. However, these effects depend on the concentration, and administration methods are different and sometimes contradictory. It can be used successfully to treat or inhibit autoimmune diseases. Although the exact mechanism of this treatment is unknown, it appears to involve inhibiting downstream intracellular pathways that lead to the secretion of pre-inflammatory cytokines. These events lead to the secretion of Th2 cytokines such as IL-10 and TGF-β and inhibit inflammation. Regarding the effect of this substance in the process of apoptosis, the results are contradictory. as this substance leads to the activation of caspase-3 and increased FASL expression at the cell surface and thus increases apoptosis(reference), while the substance leads to increased cell survival and cell proliferation (reference). The effect of this substance on the aggravation of tumors, angiogenesis and metastasis is due to the same anti-apoptotic effects of this substance. What is certain is that the concentration and method of administration and the duration of administration have great effects on the type of body responses, however, the exact effects and mechanism of this substance on the immune system must be studied extensively. Finally, it is emphasized that smoking is certainly a harmful factor for human health. Although nicotine has beneficial anti-inflammatory effects when used alone, due to its toxicity and addictive potential, further research is needed to find the specific α7nAChR agonists with few side effects.

## Author Contributions

**Conceptualization: **Leila Mahmoudzadeh.

**Data curation: **Leila Mahmoudzadeh.

**Investigation: **Seyyed Meysam Abtahi Froushani.

**Methodology: **Seyyed Meysam Abtahi Froushani.

**Project administration: **Maryam Mahmoudzadeh.

**Supervision: **Seyyed Meysam Abtahi Froushani.

**Visualization: **Marjan Ajami.

**Writing-original draft: **Leila Mahmoudzadeh.

**Writing-review&editing: **Maryam Mahmoudzadeh.

## Ethical issues

 Not applicable.

## Conflict of Interest

 Authors declare no conflict of interest in this study.
